# Multiobjective Optimization of Fabrication Parameters of Jute Fiber/Polyester Composites with Egg Shell Powder and Nanoclay Filler

**DOI:** 10.3390/molecules25235579

**Published:** 2020-11-27

**Authors:** Ganesan Karuppiah, Kailasanathan Chidambara Kuttalam, Murugesan Palaniappan, Carlo Santulli, Sivasubramanian Palanisamy

**Affiliations:** 1 Department of Mechanical Engineering, VSB College of Engineering and Technical campus, Coimbatore 642109, Tamilnadu, India; 2 Center for Materials Research, Department of Mechanical Engineering, Sethu Institute of Technology, Kariapatty 626106, Tamilnadu, India; uthrakailash@yahoo.co.in; 3 Department of Mechanical Engineering, College of Engineering, Al-Imam Mohammed Ibn Saud Islamic University, Riyadh 11432, Saudi Arabia; mpapathi@imamu.edu.sa; 4 School of Science and Technology, Università di Camerino, 62032 Camerino, Italy; carlo.santulli@unicam.it; 5 Department of Mechanical Engineering, Kalasalingam Academy of Research and Education, Anand Nagar 626128, Tamilnadu, India; sivaresearch948@gmail.com

**Keywords:** eggshell powder, nanoclay, COPRAS, jute fabric, composites, optimization

## Abstract

In the present study, a model is presented to optimize the fabrication parameters of natural fiber reinforced polyester matrix composites with dual fillers. In particular, jute fiber mat was chosen as reinforcement and eggshell powder (ESP) and montmorillonite nanoclay (NC) were selected as fillers. The weight per square meter (GSM) of the fiber, the weight percentage of ESP and NC have been chosen as independent variables and the influence of these variables on tensile, flexural and impact strength of the composite has been inspected. The permutations of the different combinations of factors are intended to accomplish higher interfacial strength with the lowest possible number of tested specimens. The experiments were designed by the Taguchi strategy and a novel multi-objective optimization technique named COPRAS (COmplex PRoportional ASsessment of alternatives) was used to determine the optimal parameter combinations. Affirmation tests were performed with the optimal parameter settings and the mechanical properties were evaluated and compared. Experimental results show that fiber GSM and eggshell powder content are significant variables that improve mechanical strength, while the nanoclay appears less important.

## 1. Introduction

The need for low-cost and environment-friendly materials has been continuously increasing, with sustainability becoming a stringent requirement. This allowed some space for natural fiber composites, especially as the replacement of fiberglass: this process developed substantially at the beginning of this century, but is deemed to continue and grow, also for the increasing availability and variety of ligno-cellulosic fibers for the purpose [1]. The selection of fibers for use in composites has normally been based on environmental together with economic considerations, e.g., the fact that the fiber is a by-product of another sector, e.g., textile, or that the local origin of the crop for fibers would result in reduced transportation costs. More recently, also mechanical data have been added to the picture to allow a selection that would eventually appear more suitable for engineering purposes: this proved particularly of interest in sectors, such as the automotive industry, with growing interest on natural fiber composites [2]. In most engineering sectors, the replacement of oil-based polymers as matrices appears still cumbersome in terms of controlled processing and performance. A major challenge for the investigator is to obtain a sufficiently strong interface between the polymer matrix, which is normally hydrophobic, and vegetable fibers, whose nature is hydrophilic: in principle, this fact impedes the adhesion between the two phases, being therefore detrimental for the composite properties. The chemical treatment of vegetable fibers, widely applied in the textile industry, improves their water resistance, stiffness and hardness, normally also removing undesired loose and unstructured ends of ligno-cellulosic material [3]. A possibility that is often applied is alkali treatment, which in the particular case of jute produces an effective shrinkage of the fibers, leading also to its higher crystallinity [4].

In case treatment is not sufficient to achieve the desired properties, also the introduction of ceramic fillers may be required. In jute, for example, silicon carbide and aluminum oxide proved beneficial on mechanical and impact properties, when introduced up to 10 wt.% in the composite [5]. However, also other options are possible, which can be cheaper, being possibly based on waste from other industries, or allow an even more significant dispersion in the laminate.

Eggshell (ESP) is mainly based on calcium carbonate (CaCO_3_). More precisely it contains 96% CaCO_3_, 1% magnesium carbonate (MgCO_3_), 1% calcium phosphate (Ca_3_PO_4_), the remaining part being formed by a natural network of sulfated polysaccharides, collagen, and distinctive polypeptides [6]. Its abundance and repeatable structure make egg shell an adaptable and free, as coming from food industry waste, source of bio-based calcium carbonate [7,8]. A crucial aspect is represented by assessing the maximum amount of egg shell powder (ESP) that can be introduced in the composite: in the case, e.g., of poly(lactic acid) (PLA), a positive effect on tensile strength and modulus is obtained with up to 4 wt% of ESP, then declines rapidly [9]. This suggests the need for a fine-tuning that, if correctly carried out, would allow obtaining a more ductile behavior and a controlled damage under loading as the effect of introducing egg shell powder as polymer filler.

Montmorillonite nanoclay (NC) also offers successful interaction and incorporation with as filler for polymer matrices [10]. This is the case also when combined with natural fibers, e.g., sisal [11]. The integration of nanoclay up to 3 wt% offered a suitable damage tolerance and increased shear properties, so to try to overcome one of the most considerable drawbacks in the use of composites [12].

This work, developing the mechanical assessment performed on a similar material system [13], aims at combining of different factors, namely eggshell powder and nanoclay content, and jute mat areal weight. The study performed in [13] mainly concentrated on the effect of alkali treatment on jute mat, although the influence of the different factors on the performance and especially of their combination was not evaluated. This is the goal of this work, which may lead to optimized values, in particular, as far as tensile strength, flexural strength, impact strength and hardness are concerned. Factors are purposely selected in a way to show the influence of small variations in their values. The idea is that, by testing only 9 of the possible 3^3^ = 27 alternatives, with the minimum possible number of samples, an optimal assessment would be able to identify the alternative, inside or outside the examined ones, which is possibly the most suitable for the characteristics required.

This is carried out using multiple criteria decision-making (MCDM) techniques, which have shown some potential for materials selection in the field of design. This potential is based on the fact that the correlation between multiple factors with different influence and often conflicting determines ultimately the properties, whose combination can be intended as the material performance for the envisaged use [14]. This method had limited use on natural fiber composites so far, for their inherent complexity and geometrical variability with respect to synthetic fiber ones [15]. A full factorial design has been recently applied on coir fiber composites, yet with short fiber reinforcement [16]. Previous attempts on thermosetting matrix composites including nanoclay with fillers, such as nanocarbon tubes, expressed the potential of Taguchi’s experimental design [17]. In particular, in this case the optimal assessment of alternatives has been elucidated using COPRAS (COmplex PRoportional ASsessment of alternatives) [18]. COPRAS has been known and used over two decades and more for multicriteria evaluation proving successful in both maximizing and minimizing criteria values [19].

## 2. Results and Discussion

### 2.1. Optimization of the Composite Using COPRAS

All experimental data collected in the four modes of testing carried out are reported in Table 1, Table 2, Table 3 and Table 4, referring to tensile, flexural, Charpy impact and Shore A hardness, respectively. Only three (instead of the prescribed five) specimens per test and per category have been carried out, which gives a total of 108 separate specimens, because these tests are intended as preparatory for the confirmatory experiment. Limiting the number of experiments responded also to the criterion of trying to assess whether this limitation would allow predicting the most suitable configuration for the final composite. Single, average and standard deviation values have been reported, while given the limited number of experiments analysis of variance was deemed not entirely appropriate, since this is indicated by the single experiments.

COPRAS examination was utilized to find the best combination of factors for higher mechanical quality, as requested by the application. This hypothesis is particularly appropriate for information with questionable and multiple (e.g., widely scattered) inputs and discrete properties, as it may be the case for materials selection [20]. The means beneath were pursued while applying COPRAS investigation, expressed in a number of subsequent steps:

1. Identification of attributes under investigation;
2. Decision matrix preparation (Parameters and Levels);
3. Decision matrix normalization;
4. Identify the weight of the attributes (equal weightage);
5. Identification of the weighted normalized matrix;
6. Identification of maximize indices (P_j_) and minimize indices (R_j_);
7. Identification of each alternatives relative weights of Q_j_ (COPRAS grade);
8. Ranking of alternatives;
9. Determination of the main effects of COPRAS grade;
10. Confirmation experiment;
11. Select the optimal values of process parameters.

Taguchi strategies employ the signal/noise (S/N) ratio to measure the reliability of the investigational results, because this proportion accounts for both the average and the variation of these outcomes. The S/N ratio accounts for the minimization of effect due to widely scattered parameters. It is computed in the light of the smaller the better using Equation (1), in the specific case applied only for hardness, and in the light of the larger the better, in the specific case applied for tensile and flexural strength and impact energy, using Equation (2).

(1) $$S/N=-10\log_{10}[\;\sum (y^{2})/n]$$

(2) $$S/N=-10\log_{10}[\sum (1/y^{2})/n]$$

where *y* = experimental data; *n* = number of experiments.

Signal to noise (S/N) ratio for experimental data corresponding to the L9 orthogonal array are displayed in Table 5.

After this, the S/N ratios are normalized by dividing each of them by their sum, as per the following equation, where m represents the S/N ratio, i the samples and j the alternatives. Since the total number of parameters investigated is 4 (tensile, flexural, Charpy impact, and Shore A hardness) and to each of these the same weight is attributed, the obtained values are multiplied by 0.25, referred to as n_ij_ and reported in Table 6.

From this, the COPRAS analysis is started, by calculating the average of the sole n_ij_ values referred to the “better when higher” parameters (tensile, flexural, Charpy impact), referred to as P_j_ and the summation of all n_ij_ values, referred to as R_j_ as clarified in Equations (3) and (4). From this, Q_j_ values are obtained, defined as:

(3) $$n_{\mathrm{ij}}=\left(\frac{m_{\mathrm{ij}}}{\sum_{i=1}^{n}m_{\mathrm{ij}}}\right)*0.25;i=1,2,3;j=1,2,3$$

(4) $$Q_{j}=P_{j}\left(\sum_{i=1}^{m}\frac{R_{j}}{R_{j}\sum_{j}^{m}\frac{1}{R_{j}}}\right)$$

and the relevant values obtained are reported in Table 7, considering that the lowest Q_j_. would represent the highest suitability for use, according to the properties required. From these results, a ranking of the nine experiments is obtained, which is reported as their variable in the two last columns as their percent difference and as the ranking position, respectively. In Table 8 the average and the max–min differences between the various parameters and levels considered are also reported.

From the above observations, the three specimens that showed the highest combined effect were samples 8, 9 and 7, in that order. All these specimens included a 46 GSM jute fabric, whilst they had a possible amount of ESP and NC equal to 0%, 1.5% or 3%. This suggested that the effect of ESP and NC was considerably minor with respect to jute fabric areal weight. To establish which could be in practice the configuration for the confirmation experiment, it needs also to be observed that by long the worst data are indicated by specimen n.1, including 3% nanoclay and 0% egg shell powder. Hence, also these values are excluded from the final composite.

To establish the nanoclay content (0% or 1.5%) and the egg shell powder one (1.5% or 3%), the following three alternatives, ranked 4, 5 and 6, are considered. The only one that does not contain the excluded quantities of 3% nanoclay and 0% egg shell powder is the n.5 with 1.5% egg shell powder and 0% nanoclay.

For the aforementioned reasons, the verification experiment was carried out with jute fabric of 46 GSM areal weight, 1.5 wt.% egg shell powder and 0 wt.% nanoclay. Furthermore, Table 8 suggests that the influence of jute fabric areal weight is much more significant than that of the other two factors, as indicated from the high difference between the levels.

### 2.2. Confirmation Experiment

**Figure 1:**
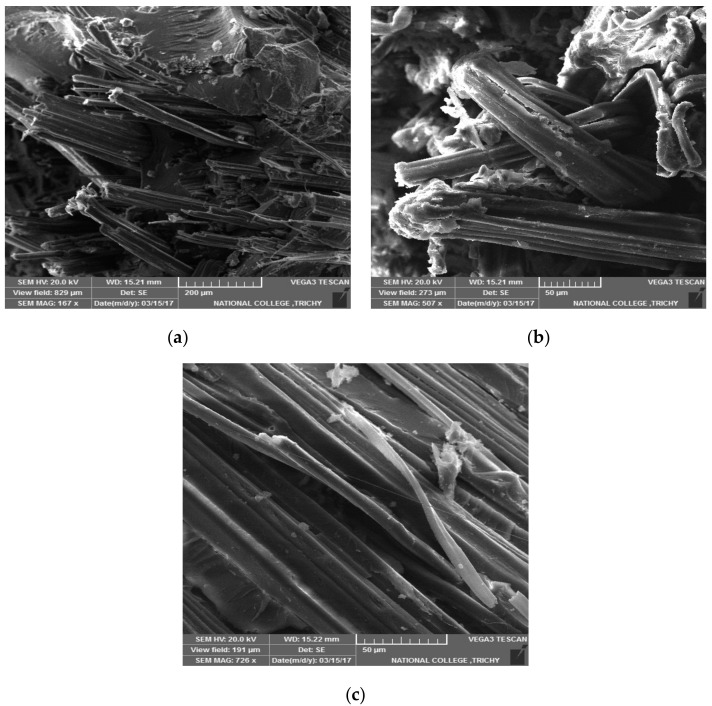
Morphological observations of the fractures by scanning electron microscopy (SEM). (**a**) Tensile fracture; (**b**) Flexural fracture; (**c**) Impact fracture.

The results of the confirmation experiment, carried out on five samples per each test, are reported in Table 9 and it can be noticed that the improvement over any of the nine initial experiments is considerable in any of the properties investigated. To complete the study, morphological investigations have been carried out using scanning electron microscope. Figure 1, showing failure obtained by: (a) tensile loading, (b) flexural loading and (c) impact loading. It generally shows that the fabric pullout phenomenon is dominant into failure. However, specific considerations can be also reported on the single testing modes. These suggest in the case of flexural loading the clear effect of fiber bending leading to pullout due to the applied load in the direction perpendicular to the composite surface. In contrast, in the case of impact loading, a series of breakage events between the matrix surface and the fabric tend to lead to decrease fabric-matrix adhesion.

The results obtained indicated that the combined effect of different ceramic fillers may be questionable. It is of course depending also on the fact that in this specific case the laminate was investigated for its easier mechanical workability (e.g., hole drilling), which required therefore achieving a lower hardness. The combination of jute fibers with nanoclay proved of interest in rubber matrix composites, which are characterized by considerable elongation [21]. This might not be the case for thermosetting polymer composites. Another significant observation is the fact that even a limited increase into areal weight of natural fiber fabric may produce a very notable effect: this is likely to occur especially for quite regular and thin fibers, such as it is the case for cottonized jute. The effect of areal weight in particular on flexural performance of natural fiber composites has also been investigated, for example for flax fiber reinforced laminates [22]. In this sense, COPRAS can represent a useful tool to better tailor the use of adequate areal weight fabric in natural fiber composites, connecting the material reinforcement characteristics to the desired properties, while avoiding excessive amounts of preliminary testing for material selection. This enabled solving conflicting trade-offs between materials, which is a possibility already explored using COPRAS, also in combination with other methods [23]. This becomes particularly crucial in the preparation of natural fiber composites, also in view of the fact that other fillers and additives are continuously proposed; these often come from secondary raw materials, such as it was the case for egg shell powder, striving to improve the sustainability of these materials.

## 3. Materials and Methods

### 3.1. Materials

Naturally woven cottonized jute mats were acquired from local shops in Coimbatore, Tamilnadu region, India. as produced by Usha Corporation Limited (UCL). These mats are normally obtained in low areal weight for possible coupling with cotton in textiles, therefore in comparable thicknesses. MMTK10 (Montmorillonite) nanoclay (surface area 220–270 m^2^/g) was supplied by Sigma–Aldrich (St. Louis, MO, USA) and Egg Shell Powder (ESP) was mashed in dimensions in the order of 30 microns, along the lines of what performed in [24]. The prepared composite contains the jute fabric mat with three slightly different areal weights (44, 45 and 46 g/m^2^). Unsaturated polyester resin was used as the matrix. Methyl ethyl ketone peroxide (MEKP) was used as the catalyst and cobalt naphthenate as the accelerator; all those chemicals were supplied by Aishwarya Polymers, Coimbatore, Tamilnadu State, India. Sodium hydroxide (NaOH) was purchased from Ponnmani chemicals, Madurai, Tamilnadu State, India.

### 3.2. Surface Modification

Detergent washing, dewaxing, and alkali treatment were carried out in light of enhancing the interfacial bonding between hydrophilic jute fabric mats to and hydrophobic polyester matrix.

In particular:

- Dirt was removed from fabrics by washing into a 5% detergent (liquid hand washing by Ariel with 5–15% non-ionic tensioactives) solution at room temperature for 10 h;
- Dewaxing, removing the traces of pectin and waxes, was ensured by keeping the fabric into 5% ethanol solution at room temperature for 1 h;
- The removal of loose fibrils and hardening the surface was achieved by keeping the fabric into a 5% NaOH solution at room temperature for 6 h, then the alkali-treated fiber mats surface was sprinkled by using a 2% acetic acid (CH_3_COOH) solution.

Each phase was followed by washing with refined water and natural drying.

### 3.3. Fabrication and Testing

The composite was set up by shifting the wt% of nano-clay eggshell powder and fiber GSM dependent on the grouping of the standard L9 orthogonal array. The fabric mat polyester resin extent is 1:3. Jute mat weighing 42 g was taken, and the form was set up using 101 g of polyester resin. The CN and MEKP were added as 1.5 wt% of the total resin weight each. For the overhaul of the scrambling, the ESP/NC polyester delays were mechanically mixed in the quick shear blender 500 r/min for around an hour at ambient temperature. After that, the mix was held in a vacuum broiler to clear the air pockets to show up in it. By then, the mold was closed by applying 100 kg/cm^2^ weight using pressure forming machine, by then the composite was re-established up to 12 h at room temperature. The fabricated composites were taken out of the mold and cut into the dimensions recommended by the ASTM D3039 standard for flexural and tensile testing and ASTM D256 for Charpy impact testing.

### 3.4. Control Factors and Matrix of Experiments

Multi criteria decision-making (MCDM) is aimed at evaluating the combination of conflicting criteria in order to take a decision, in the specific case of this work to clarify which may be the most suitable mix of values for the three factors, concerning jute, eggshell powder and nanoclay, leading to the most desirable properties for the application envisaged (automotive interior panels). Here, COPRAS technique has been used to forecast the impact of limiting criteria esteems on the last outcome, to check the computations and to consider conceivable flimsiness of evaluations yielded by the method because of the particular character of the actual data. Hence, L9 orthogonal array with three parameters (fiber size in GSM, weight percentage of eggshell powder and nanoclay) in three levels was used to optimize the variation of ESP/NC, to accomplish better tensile, impact and flexural strength with a lower hardness, able to provide an easier accommodation for the automotive panels. The control factors for three levels of COPRAS analysis were reported in Table 10, and the L9 matrix is shown in Table 11.

## 4. Conclusions

In this investigation, the influence of fabric areal weight and hardening fillers, such as egg shell powder (ESP) and montmorillonite nanoclay (NC), on the quality of jute mat strengthened unsaturated polymer composite, assessed through tensile, flexural, impact and hardness measurements, has been explored. For the enhancement of the quality, the combination of those parameters has to be optimized; an analysis which has been performed using the COPRAS based Taguchi method. The COPRAS was prescribed to overhaul various objectives into a single target work (to help the examination of the Taguchi procedure). The equal individual response weights for the parameters were allotted to determine COPRAS grade to determine the optimal combination of parameters. This combination, namely including 46 g/m^2^ jute fabric with 1.5 wt.% eggshell powders without nanoclay, has been therefore assumed to be the best one amongst possible combinations for the required conditions (improved tensile, flexural and impact strength with decreased hardness).

## Figures and Tables

**Table 1:** Experimental tensile strength (MPa) for the different specimens.

Category	Specimen 1	Specimen 2	Specimen 3	Average Value	St. Dev.
1	28.6	30.5	16.8	29	7.4
2	29.9	34.2	29.9	31.3	2.5
3	28.3	28.3	33.2	29.9	2.8
4	38.6	35.4	26.9	33.6	6.0
5	32.3	30.1	29.2	30.5	1.6
6	31.1	34.4	32.4	32.6	1.7
7	35.9	38.4	41.7	38.7	2.9
8	44.1	46.8	42.9	44.6	2.0
9	36.9	45.6	35.2	29.2	5.6

**Table 2:** Experimental flexural strength (MPa) for the different specimens.

Category	Specimen 1	Specimen 2	Specimen 3	Average Value	St. Dev.
1	47.9	60.2	62.9	57	8
2	62.9	71	97.3	77.1	18
3	74.9	56.9	68.9	66.9	9.2
4	115.7	67.8	73.7	85.7	26.1
5	81.8	69.9	87.8	79.8	9.1
6	87.8	82.8	88.8	86.5	3.2
7	88.8	94.8	85.8	89.8	4.6
8	99.6	96.4	112.6	102.9	8.6
9	74.9	69.9	76.8	73.9	3.6

**Table 3:** Experimental impact energy (J) for the different specimens.

Category	Specimen 1	Specimen 2	Specimen 3	Average Value	St. Dev.
1	0.9	0.9	0.88	0.89	0.01
2	2.08	1.73	1.08	1.63	0.51
3	1.21	1.49	1.35	1.35	0.14
4	1.88	2.24	1.63	1.58	0.31
5	1.88	1.73	1.89	1.83	0.09
6	1.93	2.34	2.02	2.12	0.22
7	2.34	1.94	1.63	1.97	0.36
8	2.72	1.98	2.56	2.09	0.39
9	2.45	2.13	2.21	2.26	0.17

**Table 4:** Experimental Shore A hardness for the different specimens.

Category	Specimen 1	Specimen 2	Specimen 3	Average Value	St. Dev.
1	95.7	92	92.2	93.3	2.1
2	93.7	90	91.8	91.8	1.9
3	75.9	83.8	80.4	80	4.0
4	87.1	85.9	84.1	85.7	1.5
5	73.3	71.2	72.7	72.4	1.1
6	77.7	75.9	72.1	75.2	2.9
7	91.3	97.7	94.8	94.4	3.2
8	80.9	80.7	80.5	80.7	0.2
9	86.3	85.7	85.9	86	0.3

**Table 5:** S/N ratio.

Category	Tensile Strength (MPa)	Flexural Strength (MPa)	Impact Strength (J)	Hardness
1	27.11	35.77	−0.97	−39.40
2	29.87	37.74	4.23	−39.26
3	29.52	36.52	2.59	−38.07
4	30.54	38.68	5.64	−38.66
5	29.70	38.06	5.25	−37.19
6	30.27	38.66	6.43	−37.53
7	31.75	39.08	5.90	−39.52
8	32.99	40.28	7.68	−38.14
9	31.87	37.38	7.09	−38.69

**Table 6:** Normalized S/N ratio.

Category	Tensile Strength (MPa)	Flexural Strength (MPa)	Impact Strength (J)	Hardness
1	0.0268	0.0261	−0.0055	0.0284
2	0.0270	0.0275	0.0241	0.0283
3	0.0267	0.0267	0.0148	0.0275
4	0.0277	0.0283	0.0322	0.0279
5	0.0269	0.0278	0.0300	0.0268
6	0.0274	0.0282	0.0367	0.0271
7	0.0288	0.0286	0.0337	0.0285
8	0.0298	0.0294	0.0438	0.0275
9	0.0289	0.0273	0.0405	0.0279

**Table 7:** COPRAS analysis.

Category	Pi	Rj	1/Rj	Qj	Nj	Rank
1	0.0159	0.0757	13.21	0.1750	72.3	9
2	0.0263	0.1071	9.33	0.1384	91.3	7
3	0.0227	0.0957	10.45	0.1483	85	8
4	0.0294	0.1160	8.62	0.1330	95	5
5	0.0282	0.1115	8.97	0.1360	92.9	6
6	0.0308	0.1194	8.38	0.1314	96.1	4
7	0.0304	0.1195	8.37	0.1302	97	3
8	0.0344	0.1306	7.66	0.1266	100	1
9	0.0322	0.1246	8.03	0.1284	98.3	2

**Table 8:** Average and max–min variation between the different factors and levels.

Parameters	Avg. L1	Avg. L2	Avg. L3	Max-Min Difference
A: Jute areal weight (JAW)	82.9	94.6	98.4	15.5
B: Eggshell powder (ESP)	88.1	94.7	93.1	6.6
C: Nanoclay (NC)	91.6	94.8	89.4	5.4

**Table 9:** Confirmation experiment results.

Parameter	Average Values and Standard Deviation
Tensile strength (MPa)	54.7 ± 5.5
Flexural strength (MPa)	109.2 ± 8.3
Impact strength (J)	3.14 ± 0.35
Shore A hardness	58.1 ± 6.9

**Table 10:** Parameters (P) and levels (L) matrix for experimental work.

Parameter	Variable	L1	L2	L3
P1	Jute fabric areal weight (g/m^2^)	44	45	46
P2	Egg shell powder (%)	0	1.5	3
P3	Nanoclay(%)	3	1.5	0

**Table 11:** Parameters (P) and levels (L) for the different specimen categories.

Category	P1	P2	P3	Jute Fabric Areal Weight (g/m^2^)	Egg Shell Powder (%)	Nanoclay (%)
1	L1	L1	L1	44	0	3
2	L1	L2	L2	44	1.5	1.5
3	L1	L3	L3	44	3	0
4	L2	L1	L2	45	0	1.5
5	L2	L2	L3	45	1.5	0
6	L2	L3	L1	45	3	3
7	L3	L1	L3	46	0	0
8	L3	L2	L1	46	1.5	3
9	L3	L3	L2	46	3	1.5
